# Effectiveness of interventions to reduce child marriage and teen pregnancy in sub-Saharan Africa: A systematic review of quantitative evidence

**DOI:** 10.3389/frph.2023.1105390

**Published:** 2023-03-31

**Authors:** Garumma Tolu Feyissa, Lemi Belay Tolu, Matiwos Soboka, Alex Ezeh

**Affiliations:** ^1^ Dornsife School of Public Health, Drexel University, Philadelphia, PA, United States; ^2^ Department of Obstetrics and Gynecology, Saint Paul Hospital Millennium Medical College, Addis Ababa, Ethiopia; ^3^ Department of Psychiatry, Jimma University, Jimma, Ethiopia

**Keywords:** teen pregnancy, early marriage, systematic review, sub-Saharan Africa (SSA), interventions

## Abstract

**Introduction:**

Child marriage and teen pregnancy have negative health, social and development consequences. Highest rates of child marriage occur in sub-Saharan Africa (SSA) and 40% of women in Western and Central Africa got married before the age of 18. This systematic review was aimed to fill a gap in evidence of effectiveness to reduce teen pregnancy and child marriage in SSA.

**Methods:**

We considered studies conducted in sub-Saharan Africa that reported on the effect of interventions on child marriage and teen pregnancy among adolescent girls for inclusion. We searched major databses and grey literature sources.

**Results:**

We included 30 articles in this review. We categorized the interventions reported in the review into five general categories: (a) Interventions aimed to build educational assets, (b) Interventions aimed to build life skills and health assets, (c) Wealth building interventions, and (d) Community dialogue. Only few interventions were consistently effective across the studies included in the review. The provision of scholarship and systematically implemented community dialogues are consistently effective across settings.

**Conclusion:**

Program designers aiming to empower adolescent girls should address environmental factors, including financial barriers and community norms. Future researchers should consider designing rigorous effectiveness and cost effectiveness studies to ensure sustainability.

**Systematic Review Registration:**

https://www.crd.york.ac.uk/prospero/, identifier: CRD42022327397.

## Introduction

The global population is projected to reach 9.71 and 10.35 billion by 2050 and 2100, respectively. Africa will account for 105% of the projected total increase in global population between 2022 and 2100. Its population will increase from 1.36 billion in 2020 to 3.92 billion by 2100. This rapid growth, over a relatively short period, together with the compositional effects it will engender, will have significant implications for development prospects in the region (1). Recognizing this reality, African Heads of State and Government devoted the year 2017 to “Harnessing the Demographic Dividend through Investments in Youth” (2), as a critical pathway to realizing the continent's aspiration for economic transformation. Yet, concrete action in realizing this strong political moment has remained muted. Ninety-one percent (91%) of this projected growth in Africa's population will be in sub-Saharan Africa (SSA).

The population growth of SSA is driven largely by high fertility and child onset of childbearing (3, 4). Apart from having more children on average than women in other regions, women in SSA generally start childbearing earlier and have very high levels of adolescent childbearing, high desired family size, and low levels of use of modern contraceptives (3–5). Highest rates of child marriage occur in sub-Saharan Africa and as high as 40% of women Western and Central Africa got married before the age of 18 (6).

Child marriage is the consequences of factors at such as low level of literacy of the girls and her parents, economic problems, gender norms and gender-based violence. Child marriage is one of the indicators of gender inequality and is an impediment against the full participation of girls in education and labor force (7). Child marriage and teen pregnancy remain obstacles against completion of secondary education and beyond. On the other hand, female education is a key predictor of fertility, especially secondary education and higher (8). Secondary and higher education may increase women's opportunities for work outside the home, their ability to contribute to decision making with their partners, and greater agency in taking action that advances their personal well-being and managing their family size (8–10). It is also beneficial in tackling the negative economic impacts of child marriage (11) and contributes to the achievement of the sustainable development goals (SDG 5) (12). More importantly, better educated women are more likely to prioritize the education of their children thereby creating inter-generational benefits (8).

Early marriage is often associated with increased risk of teen pregnancy. Teen pregnancy also increases the risk of deadly health consequences such as eclampsia, puerperal endometritis, low birth weight, preterm birth, and other complications (13). The World Health Organization recommends that marriage before the age 18 and pregnancy before the age of 20 should be reduced (14).

Targeting adolescent girls with programs that reduce the onset of pregnancies will achieve multiple development outcomes and contribute significantly to reducing fertility and population growth rate—in addition to its value to the girls, their families, communities, and society. First, there is a strong positive association between female education and onset of marriage and childbearing, fertility levels, fertility desires, independent fertility decisions (including use of contraception), and access to employment opportunities outside the home. More importantly, starting childbearing later increases the age gap between mothers and their daughters (intergenerational gap), which is seen as the second most important determinant of population growth, after fertility (number of children) (15).

Although there is emerging evidence from low- and middle-income countries that programs targeting adolescent girls with long-term follow up are likely to be effective and sustainable, there is limited evidence on what specific interventions or which aspects of complex intervention designs are effective in reducing child marriage and teen pregnancy (16).

Implementing evidence-based interventions that reduce teen pregnancies and child marriage is critical not only for the future development of the society, but also for the entire life and wellbeing of adolescents. It is, therefore, essential to synthesize the available evidence to guide future research and intervention focus. Our preliminary search found no recent systematic review that reported the effectiveness of interventions on teen pregnancy and child marriage in sub-Saharan Africa. Cognizant of this, this systematic review was aimed to identify and synthesize evidence on the effect of interventions that have been implemented to reduce teen pregnancy, and child marriage in sub-Saharan Africa.

### Research questions

The research questions addressed in this review were:

1. What is the best available evidence on the effectiveness of interventions seeking to reduce teen pregnancy among adolescent girls?
2. What is the best available evidence on the effectiveness of interventions seeking to reduce child marriage among adolescent girls?

## Methods

This systematic review was conducted using Preferred Reporting Items for Systematic Reviews and Meta-Analyses (PRISMA) (17). We conducted the review based on an a-priori protocol (Registry number CRD42022327397) (18).

### Search strategy

We searched the following databases: PubMed, EMBASE, CINAHL, Web of Science, Science Direct, Cochrane Database of Systematic Reviews (CENTRAL), 3ie database. Sources of unpublished studies and gray literature include Google, ProQuest Dissertation and Theses, and Google Scholar. The search was conducted in three phases with the aim of locating both published and unpublished studies. An initial limited search of PubMed and CINAHL was conducted. The text words contained in the titles and abstracts of relevant articles, and the index terms used to describe the articles were used to develop a full search strategy for the relevant databases (Supplementary file S1).

### Eligibility criteria

During the conduct of the review, we considered the following inclusion criteria.

#### Population

This review considered studies that targeted or measured the effect of interventions on outcomes of adolescent girls and female young adults.

#### Interventions

This review considered studies that reported on interventions designed to reduce child marriage, and/ or teen pregnancy.

#### Comparator(s)

We considered comparisons of interest that included, but not limited to, any default program, standard of care, no intervention or any other alternative intervention compared to the main intervention.

#### Outcomes

This review focused on the following outcomes: child marriage (marriage before 18 years), and teen pregnancy. For this review, to match with the definition adopted by the United Nations Child Fund (UNICEF), we defined child marriage as a marriage before the age of 18 years (19).

#### Context

Studies conducted at any levels (individual, group, organizational, policy and community levels) or in schools in urban, rural, and pastoral settings of sub-Saharan Africa were considered for inclusion.

#### Types of studies

We considered quantitative comparative studies having treatment and control groups published/reported in the English language and available online on or before January 26, 2022 (last search date) for inclusion. These include randomized controlled trials (both individual and cluster randomized trials), quasi-experimental studies having control groups. There was no further restriction on publication dates. Studies that did not have comparison group were not included in the review.

#### Study selection

Following the search, all identified citations were collated using EndNote (20) and duplicates were removed. Titles and abstracts were then screened for assessment against the inclusion criteria for the review by two reviewers. The full text of selected citations was assessed in detail against the inclusion criteria.

#### Methodological assessment

Two reviewers assessed the methodological qualities of the papers using the Joanna Briggs Institute (JBI) appraisal tools (21, 22). A third reviewer was invited where appropriate to settle disputes between primary and secondary reviewers.

#### Data extraction and synthesis

We extracted data from studies included in the review using priori developed extraction form containing study ID, country of origin, study design, context, study population, outcomes reported and results. In the case of incomplete information reported in the articles, we contacted the authors of primary studies. In addition, we have taken further information from multiple versions of reports for the same intervention (such as the grey report versions, project report versions and other versions published in peer reviewed journals).

Because of clinical and methodological heterogeneity and the lack of consistency in the reporting of outcomes across different studies, and because of variation in intensity, content and duration of the interventions, it was not feasible to conduct meta-analysis. Therefore, we reported the findings in narrative form. Where possible, we reported findings on subgroups separately for out of school adolescents, in-school adolescents, and based on age category.

## Findings of the review

**Figure 1:**
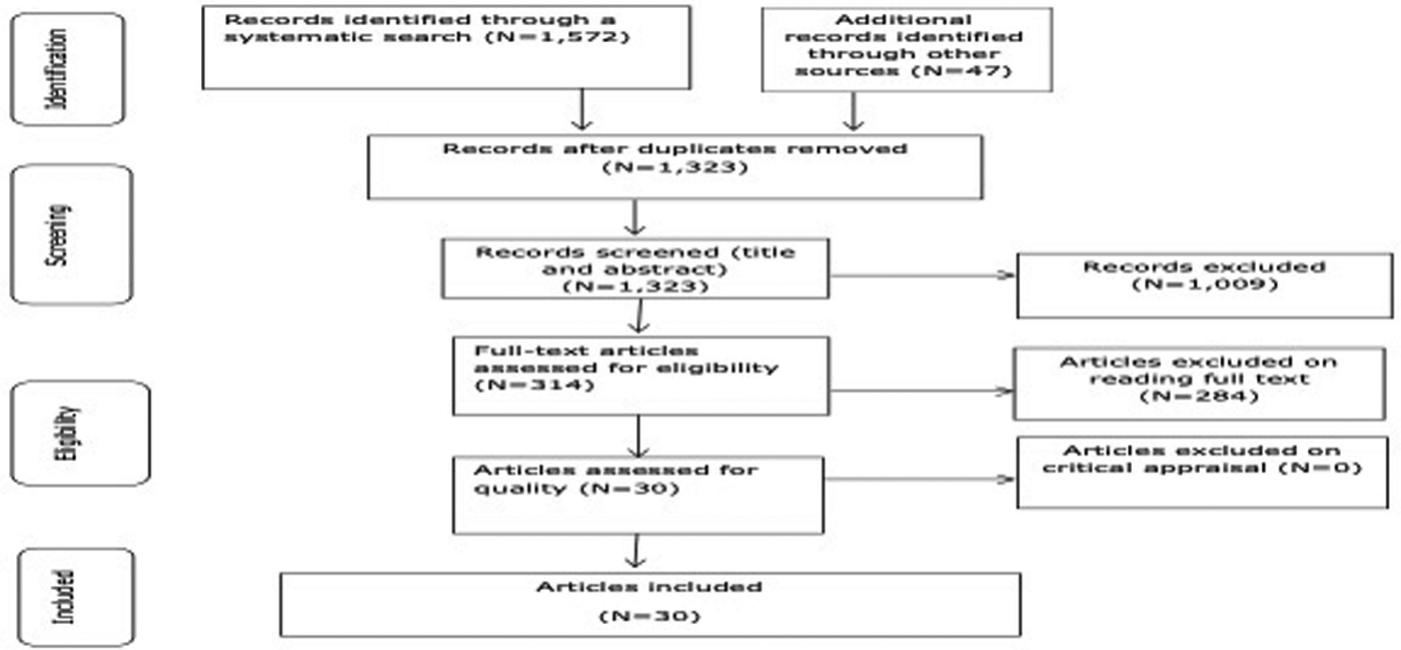
Study selection process.

Initial search yielded 1,664 articles. After removing duplicates 1,323 articles were left for screening by title and abstract, out of which 317 articles were left for full text reading. Finally, 30 articles were included. Detailed information was sought from the articles having different versions of the reports. Some of these studies had long report versions and short published versions published in peer reviewed journal articles. Even though information from the seven redundant articles was included to enrich the original studies, these redundant articles were not counted as separate studies. Hence, the records were considered as 30 articles (Figure 1). On the other hand, reports from the same trial were treated separately if they reported on different outcomes or if they addressed different data points.

The studies whose reports were merged include Baird 2009 (23), Baird 2010 (24) and Baird 2011 (25); Baird 2015 (26) and Baird 2016 (27); Duflo 2014 (28) and Duflo 2015 (29), Bandiera 2012 (30), Bandiera 2015 (31), Bandiera 2017 (32), Bandiera 2018 (33) and Bandiera 2020 (34); Dupas 2009 (35) and Dupas 2011 (36). On the other hand, four studies conducted in Zimbabwe (37, 38)) and Ghana (39, 40) that reported data at different time points from the same trial were reported separately.

### Description of the studies

Out of the 30 articles, 19 of them reported on child marriage and 22 of them on teen pregnancy, 12 of them reported on both teen pregnancy and child marriage.

Twenty-seven (27) of the studies were cluster randomized trials, three individual randomized trials (39, 41, 42), and three quasi-experimental studies (43–45). The articles covered projects conducted in nine Sub-Saharan African countries, including Zambia, Zimbabwe, Malawi, Kenya, Ghana, Uganda, Tanzania, Ethiopia, and Burkina Faso (Table 1).

**Table 1:** Characteristics of included studies

No.	Study	Country	Design	Context	Participants	Interventions arms	Results
1.	Chow 2021	Ethiopia	Quasi-experiment	School and community	Girls aged 8–17 years (901control and 1952 intervention)	**Arm 1:** Expansion (Community dialogue and information provision) **Arm 2:** Extensive (Community dialogue and information and economic incentive)	**Marriage:** Both interventions decreased the probability that a girl aged 8–17 had ever been married by 4 to 7pps. The intensive arm reduced child marriage by 2–3pps more than the expansion treatment (*p* > 0.05).
**2.**	Waidler 2022	Tanzania	CRT	Community	2,191 (1,128 control and 1,063 intervention) youth from 1,779 households	**Arm 1:** Cash (CCT and UCCT) Plus livelihoods and life skills training, and an asset transfer, with linkages to HIV and SRH services. The CCT was conditioned upon school enrolment and seeking health) **Arm 2:** Cash-only	**Marriage:** The intervention reduced probability of getting married among adolescent girls (*p* < 0.05) **Pregnancy** No significant effect on teen pregnancy(*p* > 0.05)
3.	Austrian 2022	Kenya	CRT	Urban slum community	2,147 girls aged 11–14 years in 2015 until 2019	**Arm 1:** Community dialogue for violence prevention (V) **Arm 2:** Violence prevention and Education promotion *via* conditional cash transfer, (VE) **Arm 3:** Violence prevention, education promotion, and health and life skills training (health) (VEH) **Arm 4:** Community dialogue for violence prevention, Education, Health, and financial literacy training (wealth creation) (VEHW)	**Marriage:** In the adjusted analysis, VEHW arm reduced child marriage among baseline out-of-schoolgirls, but not in the full sample (compared to V only arm) (*p* < 0.05). Both among full sample and among out-of-school girls, VE resulted in higher reduction in risk of marriage compared to V only (*p* < 0.05). No significant effect in other arms. **Pregnancy** Compared to V alone, the effects of VE on pregnancy were large and significant for girls not in school at baseline (*p* < 0.05). The effect was not significant among full sample. No significant effect on pregnancy in other arms. **Marriage and pregnancy** The pooled study arm (combination of all the three arms together (VE, VEH and VEHW) reduced marriage by 18 pp and pregnancy by 15.6pp a relative reduction of 34% and 43%, respectively (*p* < 0.05).
4.	Kengwann 2022	Kenya	RCT	Pastoral	2,075 girls 11–14 years old in 2015 until 2019	**Arm 1:** Community dialogue about inequitable gender norms and violence prevention (V) **Arm 2:** Violence prevention and education promotion *via* conditional cash transfer, (VE), **Arm 3:** VEH, Violence, Education promotion, and weekly group meetings with health and life skills training (Health) **Arm 4:** Violence, Education promotion, Health, and financial literacy training (Wealth creation) (VEHW)	**Pregnancy** No statistically significant effect of HEW, VE, VEH vs. V only both for the full sample and for those aged 13–14 years at baseline on teen pregnancy (*p* > 0.05).
5.	Erulkar 2020	Burkina Faso and Tanzânia	Quasi experimental	Rural schools and community	2,500 girls aged 12–17 years in each country	**Arm 1:** Community dialogue **Arm 2:** School supplies, **Arm 3:** Conditional livestock asset transfer conditioned on the 12–17-year-old girls remaining unmarried and in school for the duration of the pilot period (27–28 months) **Arm 4:** Combined (Community dialogue, school supplies, and conditional asset transfer) **Arm 5:** Control	**Marriage** **Burkina Faso:** Among those in the community dialogue arm, girls aged 15–17 had two-thirds less risk RR = 0.33; 95% CI, 0.19, 0.60) of being married compared to the control site. **Tanzania:** Girls aged 12–14 in the multicomponent arm had two-thirds less risk of being married (RR = 0.33; 95% CI = 0.11, 0.99), and girls 15 to 17 in the conditional asset location had half the risk (RR = 0.52; 95% CI = 0.30, 0.91).
6.	Erulkar 2009	Ethiopia	Quasi	School	Adolescent girls aged 10–19 (188 intervention and 272 girls in control)	**Arm 1: Intervention** a) Community conversations(b) School supplies (c) Conditional asset transfer, promising girls, and their families a goat if they remain unmarried and in school (d) Girls’ groups led by adult women mentors **Arm 2:** control	**Marriage** At end line, girls aged 10–14 in the intervention arm were 1/10th as likely to have gotten married compared to girls at the control site (0.1). Girls aged 15–19 in the intervention arm had 2.4 times elevated risk of getting married (*p* < 0.01)
7.	Handa 2015	Kenya	CRT	Community	1,549 females aged 12 to 24	**Arm 1: Intervention** Cash Transfer (UCCT) for OVC **Arm 2:** Control	**Marriage:** At 4 years-follow up UCCT reduced likelihood of pregnancy by 5.5 pp (*p* < 0.05) relative to control arm, no impact on child marriage (reduction by 20%) (*p* = 0.21).
8.	UNC 2016	Malawi	CRT	Household	2,109 Youth 13–19	**Arm 1: Intervention** Cash transfer (UCCT) to ultra-poor rural households **Arm 2:** Control	**Pregnancy:** After 2 years, among the 13–19-year-olds at baseline, 20% of female youth in intervention arm and 24% of female youth in control arm had experienced pregnancy(*p* > 0.05). **Marriage:** 7% of the intervention sample and 8% of the control arm reported being married (*p* > 0.05).
9.	Dake 2018	Malawi and Zambia	CRT	Rural household	Youth aged 14 to 21 years (1,023 in Malawi and 1,070 in Zambia	**Arm 1:** Intervention Cash transfer (UCCT) to ultra-poor rural households **Arm 2:** Control	**Malawi** **Marriage:** Cash transfer programs had no impact on ever married (b = −0.00428) (*p* > 0.05). **Pregnancy:** No impact on ever pregnant (0.00507) (*p* > 0.05). **Zambia** **Marriage:** No impact on ever married (b = 0.0117) (*p* > 0.05). **Pregnancy:** No impact on ever pregnant (0.000716) (*p* > 0.05).
10.	Baird 2016	Malawi	CRT	In school and out of school	Never married young women aged 13 to 22 years old	**Arm 1:** Conditional Cash transfer (CCT) (based on school attendance), to families of school aged girls and school fee waives **Arm 2:** Unconditional cash transfer (UCCT) **Arm 3:** Control	**A.Marriage** **Marriage (baseline dropouts**) Baseline dropouts who were in CCT arm were 14.0, 15.7, and 10.7 pp less likely to have been ever married at one-, two-, and five-year follow-ups, respectively (*p* < 0.01). **Marriage (baseline schoolgirls CCT):** No effects on teen child marriage for the CCT arm among baseline schoolgirls at round 4 (as in earlier rounds). **Marriage (baseline schoolgirls UCCT):** UCCT was effective on baseline schoolgirls during and immediately upon completion, but UCCT did not have any impact on ever married on baseline schoolgirls at (round 4) five-year follow up (*p* > 0.05). **B.Pregnancy** **Pregnancy (baseline dropouts):** Baseline dropouts who were in CCT arm were 5.7, 8.1, and 3.8 pp less likely to have ever been pregnant (*p* < 0.10). **Pregnancy (baseline schoolgirls CCT):** No effects on teen pregnancy for the CCT arm among baseline schoolgirls at round 4 (as in earlier rounds) (*p* > 0.05). **Baseline schoolgirls UCCT:** UCCT was effective on baseline schoolgirls during and immediately upon completion, but UCCT did not have any impact on ever pregnant on baseline schoolgirls at (round 4) five-year follow up (*p* > 0.05).
11.	Baird 2009						**Marriage** **During the program**: 4.3% were married in the control group. Marriage rates were unchanged in the CCT arm but significantly lower in the UCT arm. **Immediately after the program ended):** The prevalence of marriage rose to 18.0% in the control arm with an insignificant reduction of 1.2 pp in the CCT arm and a significant reduction of 7.9 pp (44%) in the UCT arm. The likelihood of ever being pregnant was reduced by 6.7pp (27%) in the UCT arm (*p* < 0.01)
12.	Bandiera 2015	Uganda	CRT	Clubs in fixed meeting places in communities (Rural and urban)	5,966 Girls aged 14–20 (3,964 in treatment 2,002 in control)	**Arm 1:** Intervention (Empowerment and livelihood program for adolescents implemented *via* adolescent development clubs, female mentor (life skills training to run small-scale enterprises, and vocational skills training, information on sex, reproduction, and marriage **Arm 2:** Control	**Marriage:** Two years after intervention, girls in intervention arm were 6.9 pp less likely to be married, corresponding to 53% of the baseline mean. In control arm, marriage rates rose naturally from 12% to 18% from baseline to follow-up (*p* < 0.05). **Pregnancy:** Teen pregnancy fell by 26% in the intervention arm (*p* < 0.05).
13.	Bandiera 2020						
							**Marriage:** At endline (four years after intervention) participants are 8pp less likely to be married. Marriage fell by 62% (*p* < 0.05). **Pregnancy: T**he probability of teen pregnancy was 3.8pp lower than control (corresponding to 34% of baseline rate of pregnancy) (*p* < 0.05)
14.	Hedgayhl 2021	Zambia	CRT	School	4,922 Girls from grade 7 in 157 selected schools	**Arm 1:** Economic support alone (cash transfers to girls and their parents and payment of school fees for girls continuing to secondary school) **Arm 2:** Economic support in combination with a community dialogue (community and youth meetings to enhance sexual and reproductive health knowledge and supportive community norm) **Arm 3:** Control	**Marriage:** Compared to control, incidence of marriage before age 18 was 21% and 14% lower in the combined and economic arms, respectively CI = (combined vs. control HR 0.79, 95% CI, 0.61–1.04; economic vs. control HR 0.86, 95% CI, 0.65–1.13)
15.	Sinsamala 2021	Zambia	CRT	Rural primary (Grade 1–7) and junior secondary (Grade 8–9) schools	4,922 girls	**Arm1:** Combined Economic support to parents, girls, and community intervention **Arm 2:** Economic support only. **Arm 3:** Control	**Pregnancy:** The difference in pregnancy incidence between the combined intervention and control arm was 17% (HR 0.83 (95% CI, 0.68–1.00) and between economic support and control was 16% (HR 0.84 (95% CI, 0.69–1.02). There was no difference between the combined intervention and economic support on pregnancy incidence (HR 0.99 (95% CI, 0.83–1.17).
16.	Austrian 2020	Zambia	CRT	Community	4,661 unmarried girls aged 10–19 years	**Arm 1:** Safe spaces only **Arm 2:** Safe spaces plus health voucher **Arm 3:** Safe spaces, health voucher, financial education and saving account **Arm 4:** Control	**Pregnancy:** No positive program impacts, neither at the end of the intervention, nor two years after the end of the intervention, for ever been pregnant. **Marriage**: No positive program impacts, neither at the end of the intervention nor two years after the end of the intervention.
17.	Hallfors 2015	Zimbabwe	CRT	25 rural primary schools	328 orphan adolescent girls in grade 6	**Arm 1:** School subsidies (fees, uniforms, supplies and a school-based female helper to monitor attendance) **Arm 2:** Delayed intervention	**Marriage:** Fewer girls among the intervention arm reported marriage [18 (11.2%)] vs. [29 (23.6%)] OR = 0.37 (95%CI, 0.16–0.88) (*p* < 0.05). **Pregnancy:** Fewer girls among the intervention arm reported pregnancy [19 (11.8%) vs. [28 (22.2%)] compared to the delayed partial intervention arm after 5 years (*p* < 0.05).
18.	Hallfors 2011						**Marriage:** After two years, the control arm was much more likely to marry (OR = 2.9; CI = 1.0–8.3) compared to the intervention arm.
19.	Duflo 2010	Kenya	CRT	School	18,000 students	**Arm 1:** Teachers’ training (TT) on HIV curriculum **Arm 2:** Free school uniforms	**Marriage:** 2.5 years after baseline, girls who received uniforms were 1.7 pp less (14%) likely to be married. Both TT and the combined (uniform and TT) did not have significant difference from control (*p* > 0.05). **Pregnancy:** 2.5 years after baseline, girls who received uniforms were 1.5pp less (10%) less likely to be pregnant. Both TT and the combined (uniform and TT) did not have significant difference from control (*p* > 0.05).
20.	Duflo 2006	Kenya	CRT	School	70,000 students from 328 primary schools	**Arm 1:** Teachers’ training (TT) on HIV/AIDS curriculum **Arm 2:** Debate and write essay on role of condoms **Arm 3:** Informing about risk of HIV **Arm 4:** Reducing cost of education by providing free uniforms	**Marriage:** Girls in schools where free uniforms were provided were 1.4 pp (12%) less likely to be married (*p* < 0.05). No effect of TT. **Pregnancy:** A year after the intervention, girls who had received information on the profile of HIV infections by age and gender were 65% less likely to have gotten pregnant by adult partners (*p* < 0.05). Overall, childbearing rates fell by 32% among girls who were exposed to the information on relative risks. No effect of TT.
21.	Duflo 2015	Kenya	CRT	328 schools in Kenya's Western Province	19,000 students enrolled in grade 6 in 2,003	**Arm 1:** Educational subsidies **Arm 2:** HIV education and information (abstinence until marriage) **Arm 3:** Educational subsidy plus HIV education **Arm 4:** Add on HIV education, educational subsidy plus information on condom	**Pregnancy:** After 3 years, education subsidies reduced adolescent girls’ pregnancy (from 16% to 13%). By year 7, there was still a 7% gap in the childbearing rate between girls exposed to the education subsidy program and those in the control group (46% vs. 49%). Arm 2 did not significantly reduce teenage pregnancy. When the two programs were implemented jointly, teen pregnancy fell less than when the education subsidy was provided alone (*p* > 0.05).
22.	Dupas 2011	Kenya	CRT	328 primary schools in rural districts	13,000 youths	**Arm 1:** Teacher training (TT) on national policy on HIV/AIDS curriculum (abstinence-only (163 schools), **Arm 2:** Relative risk (RR) Communication (provision of info for Grade 8 students in 71 schools on HIV prevalence by sex and age)	**Pregnancy:** The RR reduced risk of pregnancy by 27.7% The RR program averts 29.4 pregnancies by older partners. The incidence of cross generational pregnancies declined by 61.7% in the RR arm relative to the comparison arm (*p* < 0.05). The national abstinence-only HIV education curriculum had no impact on teen pregnancy (11.1% reduction only) (*p* > 0.05).
23.	Dupas 2006	Kenya	CRT	328 primary schools in rural districts	13,000 youths	**Arm1:** Relative risk information on the prevalence of HIV disaggregated by age and gender **Arm 2:** Teachers training on school-based HIV curriculum (Control)	The RR reduced teen pregnancy by 31.4%. There was 65% reduction in the incidence of pregnancies by adult partners relative to the control arm *p* < 0.05).
24.	Asingwire 2019	Uganda	CRT	Districts	5,012 youth aged 15–24 years	**Arm 1:** Intensified YFS (distribution of fliers, video clips, SMS, IEC/SBCC, counseling, FP distribution for free) **Arm 2:** Control	Higher reduction of the probability of pregnancy in the intervention arm compared to control arm (*p* < 0.05).
25.	Doyle 2010	Tanzania	CRT	Rural community, health facilities y and schools	Adolescents (Young people who attended year 6, 7 and 8 schools)	**Arm 1:** School SRHE (by teachers, participatory drama), YFRHS *via* education of health workers, condom promotion and community activities **Arm 2:** Control	Nearly 102/3,276 (3%) girls in the intervention arm compared to 91/3,238 (3%) in the comparison arm (AOR 1.09, 95% CI, 0.94, 1.26) (*p* > 0.05).
26.	Cowan 2010	Zimbabwe	CRT	30 communities in 7 districts in South-Eastern Zimbabwe	4,684 youth aged 8–22 years	**Arm 1:** Community-based multi-component HIV prevention intervention delivered to young people, parents, and clinic staff and was theoretically based in social learning theory **Arm 2:** Control	No impact of the intervention on pregnancy rate, which was 5.9% in control Vs 5.3% in intervention arm AOR 0.83 [0.50–1.35] Adjusted for *a priori* confounders (age, strata, marital status & education)]. Unmarried women in intervention arm had lower risk becoming pregnant (AOR 0.55, 95% CI, 0.32–0.95) than those in control arm.
27.	Dunbar 2014	Zimbabwe	RCT	Out-of- school high density urban area	315 out-of-school adolescent female orphans (having lost at least one parent) aged 16–19	**Arm 1:** Life-skills and health education, vocational training, micro-grants (in the form of capital equipment, supplies or additional training) and social supports) **Arm 2:** Life-skills and health education alone	Fewer unintended pregnancies among intervention participants [HR 0.61, 95% CI (0.37, 1.01)]
28.	Rokicki 2017	Ghana	CRT	38 secondary Schools in Accra	756 females students aged 14 to 24 years	**Arm1:** Unidirectional intervention **(**text message sent participants text messages with reproductive health information. **Arm 2:** Interactive intervention (engaging adolescents in text messaging reproductive health quizzes. **Arm 3:** Control.	Both unidirectional [odds ratio (OR) = 0.14; 95% CI = 0.03, 0.71] and interactive interventions (OR = 0.15; 95% CI = 0.03, 0.86) lowered odds of self-reported pregnancy for sexually active participants (at 15 months follow up)
29.	Duflo 2021	Ghana	RCT	2,064 students (1,036 females).	Secondary school	**Arm 1:** Scholarship (tuition and exam fee **Arm 2:** Control	**Marriage:** 12 years after the intervention, the likelihood of getting married reduced by 0.096 (*p* = 0.08) compared to the control mean of 0.53. In 2019, the probability of getting married reduced by 0.062 (*p* = 0.067) compared to the control mean of 0.475. **Pregnancy:** Scholarship recipients were 0.07(*p* = 0.037) less likely to have ever been pregnant compared to comparison mean of 0.479.
30.	Dupas 2017						
							**Marriage:** Eight years after the intervention, there was 9.1pps less probability of living with partner at age 25 (compared to mean control 0.344). **Pregnancy:** Scholarship recipients were 10.7pp (18%) less likely to have ever been pregnant (compared to control mean of 0.583) (*p* < 0.01)

**NB:** AOR, Adjusted odds ratio; CCT, Conditional cash transfer; CI, Confidence interval; HR, Hazard ratio; IEC, Information, education and communication; OVC, Orphan and vulnerable children; PP, percentage point; RR, Relative risk; SBCC, Social and behavioral change communication; SRH, Sexual and Reproductive Health; TT, Teachers’ training; UCCT, Unconditional cash transfer; V, Violence prevention; VE, Violence prevention combined with and education promotion; VEH, Violence, Education and Health; VEHW, Violence prevention, education promotion, health, and wealth creation; YFRHS, Youth friendly reproductive health services; YFS, Youth friendly services.

#### The methodological quality of the included articles

The appraisal scores of the randomized controlled trials and cluster randomized trials are shown in Appendix 1 and 2 respectively. The scores for the individual and cluster randomized trials ranged from 9/13 to 10/13. Almost all studies could not blind participants, researcher, and data collectors, which is also expected given the nature of the interventions. In addition, few of the studies reported baseline imbalances. In the case of quasi-experimental studies included in this review, the main risk of bias was related to the comparability of the groups even though the primary authors have attempted to account for this effect using analysis.

## Effectiveness of the intervention on child marriage

The interventions reported in the included papers are broadly classified into one the following categories based on the areas they addressed (health, education, wealth, or community dialogue).

a. Interventions aimed to build educational assets (E),
b. Interventions aimed to build life skills and health assets (H),
c. Interventions aimed to build livelihood and/or financial skills (Wealth building interventions (W),
d. Interventions aimed to change community norms (Community dialogues to change community norms about women empowerment including gender violence or child marriage or both (C),
e. Combination of one or more of the above.

As such some of the projects have addressed more than one of the educational, health and livelihood and community dialogue components. Some comprised four components, some consisted of three components, and others consisted of two components (Table 2). Note that some studies were multi-arm designs, and they simultaneously reported the effect of one component, two component, three component and four component interventions. Community dialogue designed for violence prevention has been used both as control and combined with multi-component interventions. It is critical to note that the studies the reported one, two, three and four component interventions were not mutually exclusive, because a single study with multiple arms may report different combinations of interventions and thus contributing to one, two, three and four component interventions. In addition, when we refer to “component”, we are referring from the perspective of the broader category even though there are narrow multiple interventions within one broad component intervention. For instance, a one component heath intervention may contain training and information on sexual and reproductive health, menstrual hygiene, HIV/AIDS, providing clinic vouchers, etc.

A. Programs/Projects that addressed four components (Health, Education, Wealth, and community dialogue)

**Table 2:** Effectiveness of different categories of interventions on child marriage across the different subgroups and settings.

Intervention type	Study ID	Country	10–19	Ages 8–17	Ages 14–19	Ages l4 years or less	Ages 15–19	Orphans	Out of school	School Adolescents	Ultra-poor households
**Four component comprehensive interventions**											
Violence prevention, health, and educational and wealth promotion	Austrian 2022	Kenya	** **	** **	Not effective	** **	** **	** **	**Effective**		** **
Education promotion (*via* CCT), wealth, health promotion plus community dialogue	Hegdahl 2021	Zambia			** **	** **	** **	** **	** **	Not effective	** **
Community dialogue and information on FP and SRH *via* school clubs plus economic incentive in the form of school materials and revolving funds (HEWC)	Chow 2021	Ethiopia		Effective	** **	** **	** **	** **	** **		** **
**Three component comprehensive interventions**											
(UCCT plus CCT PLUS livelihoods and life skills training, mentoring and an asset transfer, with linkages to strengthened government-run HIV and SRH services)	Waidler 2022	Tanzania	** **	** **	Effective (among females)	** **	** **	** **	** **	** **	** **
Violence prevention, health, and educational promotion	Austrian 2022	Kenya	** **	** **	Not effective	** **	** **	** **	Not effective		** **
Combined (school promotion, asset transfer and community dialogue)-HEWC	Erulkar 2020	Tanzania	** **	** **		Effective	Not effective	** **	** **	** **	** **
	Erulkar 2009	Ethiopia	** **	** **		Effective	Reverse effect	** **	** **	** **	** **
**Two components**											
Violence prevention, and education promotion	Austrian 2022	Kenya		** **	Effective	** **	** **	** **	**E**ffective		** **
Empowerment and livelihood and life skills training and RH information (Health and Wealth)	Bandiera 2020	Uganda	Effective	** **	** **	** **	** **	** **	** **	** **	** **
Community dialogue plus information on FP and SRH *via* school clubs (Health and community dialogue)	Chow 2021	Ethiopia		Effective	** **	** **	** **	** **	** **	** **	** **
Social (safe space), health voucher, and wealth (saving account) intervention	Austrian 2020	Zambia		Not effective	** **	** **	** **	** **	** **		** **
Social (safe space) and wealth (saving account) intervention	Austrian 2020	Zambia		Not effective	** **	** **	** **	** **	** **		** **
Add on HIV education, educational subsidy plus information on condom	Dulfo 2015	Kenya			** **	** **	** **	** **	** **	Not effective	** **
Educational subsidy plus Abstinence only HIV curriculum	Duflo 2010	Kenya			** **	** **	** **	** **	** **	Not effective	** **
Tuition, material, and economic support (Education and Wealth)	Hegdahl 2021	Zambia			** **	** **	** **	** **	** **	Not effective	** **
**Single component**											
Community dialogue	Erulkar 2020	Burkina Faso					Effective				
	Erulkar 2020	Tanzania				Not effective	Not effective				
Social (safe space) only intervention	Austrian 2020	Zambia		Not effective							
Education promotion (*via* CCT)	Erulkar 2020	Burkina Faso					Not effective				
	Erulkar 2020	Tanzania				Not effective	Not effective				
	Hallfors 2011	Zimbabwe						Effective			
	Hallfors 2015	Zimbabwe						Effective			
Educational subsidy	Dulfo 2015 and Duflo 2010	Kenya								Effective	
	Duflo 2021	Ghana								Effective	
Abstinence only HIV education	Dulfo 2015 and Duflo 2010	Kenya								Not effective	
Conditional asset Transfer alone	Erulkar 2020	Tanzania				Not Effective	Not Effective				
Conditional cash transfer- short-term (<2 years)	Baird 2010	Malawi							Effective	Not Effective	
CCT-5 year follow up	Baird 2016	Malawi							Effective	Not effective	
UCCT-short term (≤2 years)	Baird 2010	Malawi							-	Effective	
UCCT-Long term (5 years)	Baird 2016	Malawi							-	Not effective	
Cash Transfer (UCCT) for OVC-long term (4 year)	Handa 2015	Kenya						Not effective			
UCCT for ultra-poor (2 years	UNC 2016	Malawi									Not effective
UCCT for ultra-poor-30 months in Malawi and 36 months in Zambia	Dake 2018	Malawi and Zambia (14–21 years)									Not effective

**NB**: CCT, Conditional cash transfer; FP, Family planning; HEWC, Health, education, wealth, and community dialogue; HIV, Human immune-deficiency virus; SRH, Sexual and Reproductive Health; UCCT, Unconditional cash transfer.

Out of the studies that comprised four component intervention, three of them reported on marriage outcomes. These projects were conducted in Kenya (46), Ethiopia (45) and Zambia (47). Surprisingly, the effect of these comprehensive interventions comprising violence prevention, heath, education, and wealth components, was not significant when compared to single component intervention (violence prevention) alone. An arm with four component interventions (health, education, wealth, and violence prevention) was not significantly different from an arm with single intervention (violence prevention alone). On the other hand, subgroup analysis indicates that the four-component intervention (intervention with health, education, wealth, and violence prevention) significantly reduced child marriage among baseline out-of-schoolgirls when compared to an arm with violence prevention alone.

Similarly, study conducted in Zambia reported on intervention that comprised health, education and wealth components and community dialogue to change norms around child marriage. The study reported that incidence of marriage before the age of 18 was 21% lower among the arm exposed to combinations of intervention that comprised economic support for families and tuition and material support for girls, and sexual and reproductive health education when compared to the control arm with no intervention. This change was not statistically significant (47).

An Ethiopian study that examined the effect of combined economic intervention, information and community dialogue to change norms around child marriage reported significant effect of the intervention on child marriage among girls aged 8–17 years old (45).

B. Programs/Projects that addressed three components

Studies that lie under this category included report on effect of any three combinations of Health/social, Wealth, Education, or Community dialogue. Among the included studies that reported on child marriage, four of them reported on the effect of three component interventions. These projects were conducted in Ethiopia (44), Kenya (46), Tanzania (48). One study was a multicounty study conducted in Tanzania and Burkina Faso (43). The effect of these multiple component interventions comprising violence prevention, education and health component was not significantly different from that of control arm [standalone (violence prevention) intervention] (46).

On the other hand, combined interventions comprising school promotion, asset transfer and community dialogue were effective among younger adolescents (less than 14 years of age) in Tanzania (43) and in Ethiopia (44). However, in Ethiopia, there was reverse effect among girls aged 15–19 years. Girls in treatment arm had 2.4 times higher likelihood of getting married compared to those girls in the control arm (44).

Tanzanian study reported that girls in an arm with four component interventions comprising “Cash PLUS” interventions (cash transfer plus livelihoods and life skills training, mentoring and an asset transfer, combined with linkages to strengthened government-run HIV and SRH services), were less likely to enter into marriage compared to the girls in the “Cash ONLY” arm (arm that received the cash transfer program alone) (48). The cash transfer involved both unconditional (to reduce vulnerability and increase income) and conditional (up on school enrolment or seeking essential health services).

C. Programs/Projects that comprised two components

The five studies included under this category were conducted in Zambia (47, 49), Uganda (34), Kenya (29) and Ethiopia (45). Some of these interventions focused on health/social and livelihood/wealth (34); some were focused on health/social and educational support (47); and others were focused in creating safe spaces and providing health vouchers (49). Some of these interventions were effective (34, 46) and some were not (47).

For instance, a four year follow up study conducted in Uganda reported a significant effect of empowerment and livelihood and life skills training and provision of reproductive health (RH) information (8 pp lower) compared to control communities that did not receive any of the interventions (34). A Kenyan study found that violence prevention combined with education promotion was more effective (with 6.2 pp less) when compared to violence prevention alone in reducing child marriage among out of school adolescent girls even though the effect was not significant for school adolescent girls (46). In Kenya, even though education subsidies were effective when implemented alone, they were not effective when combined with national HIV/AIDS curriculum which focuses on abstinence only. In addition, education subsidies when combined with an add on HIV education, and information on condom were not effective in reducing teen pregnancy (29).

Study conducted in Zambia reported that multicomponent intervention comprising health/social and wealth components (the provision of safe spaces for adolescents combined with heath vouchers and saving accounts) did not have any impact on child marriage when compared to control arm (no intervention) (49).

Study conducted in Zambia reported that intervention that comprised education and wealth components (financial support to families and girls, tuition, and material support) reduced incidence of marriage before the age of 18 by 14% when compared to the control arm with no intervention. This difference was not statistically significant (47).

Study conducted in Ethiopia that examined the effect of information and community dialogue to change norms around child marriage reported significant effect of the intervention on child marriage among girls aged 8–17 years old. In the study, the community dialogue was complemented by the information provided at school clubs on family planning and sexual and reproductive health issues. The approach of community mobilization consisted of training influential community members such as religious leaders, teachers, gender activists, community leaders. These influential community members would then facilitate community conversations to reflect on challenges that girls face when entering marriage and elicit empathy and dispel myths around child marriage (45).

D. Projects with one component interventions

Some of the studies reported above have also provided a report for the arms with single component interventions. Nine of the included studies reported on single component interventions (interventions addressing only either of education, wealth, heath/social and violence prevention categories). Interventions reported under this category include community dialogue (43), educational support (27, 37–39), educational subsidies, HIV/AIDS curriculum and livelihood support (UCCT) (27, 50, 51), creating safe spaces for adolescents (49).

Particularly impressive result reported from studies under this category is one Ghanaian study with relatively longer duration of follow up (39). The study reported significant impact of providing scholarship on reducing the probability of ever getting married or living with partners across years of follow up (39, 40). A multicounty study conducted in Burkina Faso and Tanzania reported that community dialogue alone was effective in reducing child marriage in Burkina Faso [girls in community dialogue arm aged 15–17 years had two-thirds less risk (RR = 0.33; 95% CI, 0.19, 0.60) of being married]. The same study reported that the community dialogue does not have significant effect on child marriage on both girls aged 12–14 years and 15–17 years old in Tanzania (43). The difference in the effectiveness of the intervention in the two countries may related to the intensity and the approaches of the interventions. The approach utilized in Burkina Faso was recruiting and training community members and training them for five days so that they will mobilize and group the community into groups of 30 people. The groups were provided 16 sessions of training on negative impacts of child marriage and the value of girls' education, after which they will devise and implement solutions. Some of the devised strategies include house-to-house campaigns and rewards or punishments to the community members. On the other hand, in Tanzania the approach included recruiting religious leaders and training them for two days on benefits of girls “education and benefits of delaying child marriage. The leaders were also trained on how to facilitate discussions and deliver messages letting them deliver messages on routine community meetings such as religious meetings. In addition, in Tanzania, there was no system for sustained contact system to trace the work of the community leaders. The community leaders were not expected to report the community members whom they contacted (43).

Study conducted in Zimbabwe demonstrated that supporting orphans and vulnerable adolescents to remain in school reduced child marriage both in the short term (2 years) (37) and long-term (5 years) follow up (38). The school support covered tuition fees, uniforms, school supplies and assigning helper to monitor participants' school attendance.

Study conducted in Malawi reported that there is no significant effect of conditional cash transfer (CCT) on marriage among schoolgirls both in short-term (2 years follow up) and long-term (5 years) follow up. The same study reported statistically significant effect of conditional cash transfer (CCT) among out-of-school girls both in short-term and long-term evaluation. The study also reported that UCCT was effective on baseline schoolgirls during and immediately upon completion, but not in the long-term (after 5 years) (27).

Using data from a four-year follow up study, a Kenyan study reported that UCCT provided to orphan and vulnerable children has no significant impact on child marriage (50). Similarly, a Malawian study reported that unconditional Cash transfer (UCCT) to ultra-poor rural households has no significant impact on child marriage (52). Other multi-country study also reported that unconditional Cash transfer (UCCT) to ultra-poor rural households did not significantly reduce child marriage both in Malawi and Zambia (51).

Study from Zambia reported that the provision of safe spaces for adolescents when provided alone or when combined with heath vouchers did not have any impact on child marriage when compared to control arm (no intervention) (49).

## Effectiveness of the intervention on teen pregnancy

The categories of interventions whose effect on teen pregnancy was reported using categories just like that of child marriage described above.

A. Programs/Projects that addressed four components (Health, Education, Wealth, and community dialogue)

Studies under these categories comprised interventions addressing combinations of health, education, wealth, and violence prevention. All the three studies comprising four component interventions reported on teen pregnancy. These projects were conducted in Kenya (42, 46) and Zambia (53). Surprisingly, the effect of these comprehensive interventions comprising combinations of health, education, wealth, and violence prevention was not significant when compared to control arm (violence prevention intervention alone (42, 46). Similarly, study conducted in Zambia reported on intervention that comprised health, education and wealth components and community dialogue to change norms around child marriage. The study found no significant effect of the multicomponent intervention on teen pregnancy compared to control arm. The combinations of intervention comprised economic support for families and tuition and material support for girls, and sexual and reproductive health education (53) (Table 3).

B. Programs/Projects that addressed three components (any three combinations of Health/social, Wealth, Education, or violence prevention)

**Table 3:** Effectiveness of different categories of interventions on teen pregnancy across the different subgroups and settings.

Intervention type	Study ID	Country	15–24	Ages 8–17	Ages 14–19	Ages 13–14	Ages 15–17	Orphans	Out of school	School Adolescents	Ultra-poor households
**Four component comprehensive interventions**											
Violence prevention, health, and educational and wealth promotion-HEWC	Austrian 2022	Kenya	** **	** **	Not effective	** **	** **	** **	Not effective	** **	** **
	Kangwann 2022	Kenya	** **	** **	Not effective	Not effective	** **	** **		** **	** **
Education promotion (*via* CCT), wealth, health promotion and life skills plus community dialogue]-HEWC	Sinsamala, 2021	Zambia	** **	** **			** **	** **	** **	Not effective	** **
**Three component comprehensive interventions**											
Cash transfer (UCCT plus CCT (conditioned up on school enrolment and health service utilization) PLUS livelihoods and life skills training, mentoring and an asset transfer, with linkages to strengthened government-run HIV and SRH services)-HEW	Waidler 2022	Tanzania	** **	** **	Not effective		** **	** **		** **	** **
Violence prevention, health, and educational promotion-HEC	Austrian 2022	Kenya	** **	** **	Not effective		** **	** **	Not effective	** **	** **
	Kangwann 2022	Kenya	** **	** **	Not effective	Not effective	** **	** **	** **	** **	** **
Life-skills and health education (HS) and vocational training and micro-grants (W)	Dunbar 2014	Zimbabwe	** **				** **	** **	Marginal effect	** **	** **
**Two components**											
Violence prevention, and education promotion	Austrian 2022	Kenya		** **	Marginally effective	** **	** **	** **	Effective	** **	** **
	Kangwann 2022	Kenya	** **	** **	Not effective	Not effective	** **	** **	** **	** **	** **
Empowerment and livelihood and life skills training and RH information (Health and Wealth)	Bandiera 2020	Uganda	Effective	** **			** **	** **	** **	** **	** **
Add on HIV education, educational subsidy plus information on condom	Duflo 2015 and Duflo 2010	Kenya	** **				** **	** **	** **	Not effective	** **
Joint HIV/AIDS curriculum (abstinence only) and provision of uniform (education subsidy)	Duflo 2015, Duflo 2010	Kenya	** **				** **	** **	** **	Not effective	** **
Social (safe space) and wealth (saving account) intervention	Austrian 2020	Zambia	** **	Not effective			** **	** **	** **	** **	** **
Tuition, material, and economic support (Education and Wealth)	Sinsamala 2021	Zambia	** **				** **	** **	** **	Not effective	** **
**Single component**											
School subsidies (fees, uniforms, supplies and a school-based female helper to monitor attendance)	Hallfors 2015	Zimbabwe						Effective			
Reducing cost of education by providing free uniforms	Duflo 2006, Duflo 2014	Kenya								Effective	
Covering tuition and exam fees	Duflo 2021	Ghana								Effective	
HIV education (abstinence only)	Duflo 2015	Kenya								Not effective	
HIV education (abstinence only) combined with educational subsidy	Duflo 2015	Kenya								Not effective	
School-based SRHE	Doyle 2010	Tanzania								Not effective	
CCT: Short-term (2years)	Baird 2016, Baird 2009	Malawi							Marginally significant effect	Not effective	
CCT: long-term (5 years)	Baird 2016	Malawi							Marginally effective (*p* < 0.1)	Not effective	
UCCT-short term	Baird 2016, Baird 2011	Malawi								Effective	
UCCT-long term	Baird 2016	Malawi								Not effective	
Cash transfer (UCCT plus CCT) PLUS livelihoods and life skills training, mentoring and an asset transfer, with linkages to strengthened government-run HIV and SRH services)	Waidler 2022	Tanzania			Not effective						
Conditional cash transfer- short-term (<2 years)	Baird 2010	Malawi							Effective	Not Effective	
CCT-5 year follow up	Baird 2016	Malawi							Effective	Not effective	
UCCT-short term (≤2 years)	Baird 2010	Malawi							-	Effective	
UCCT-Long term (5 years)	Baird 2016	Malawi							-	Not effective	
Cash Transfer (UCCT) for OVC-long term (4 year)	Handa 2015	Kenya						Effective			
UCCT for ultra-poor (2 years	UNC 2016	Malawi									Not effective
UCCT for ultra-poor-30 months in Malawi and 36 months in Zambia	Dake 2018	Malawi and Zambia									Not effective
Intensifying SRH services	Asingwire 2019	Uganda	Effective								
Social (safe space) only intervention	Austrian 2020	Zambia		Not effective							
School SRHE (led by teachers, participatory drama), YFRHS *via* education of HCWs, condom promotion and community activities to create supportive environments	Doyle 2010	Tanzania								Not effective	
HIV relative risk (RR) communication by age	Dupas 2011, Duflo 2006	Kenya								Effective	
HIV/AIDS curriculum (abstinence only)	Dupas 2011	Kenya								Not effective	
Community-based multi-component HIV prevention	Cowan 2010	Zimbabwe	Not effective								
Text messaging reproductive health information	Rcokicki 2017	Ghana	Effective								

**NB**: CCT, Conditional cash transfer; FP, Family planning; HEWC, Health, education, wealth, and community dialogue; HIV/AIDS, Human immune-deficiency virus and acquired immunodeficiency syndrome; SRH, Sexual and Reproductive Health; UCCT, Unconditional cash transfer.

Interventions were considered effective if *p* < 0.05, marginally effective if 0.05 < *p* < 0.1 and not effective if *p* > 0.1 and reverse effect if the intervention resulted in increment in teen pregnancy or early marriage relative to control arm.

Three studies reported on interventions that addressed three component interventions that addressed any combinations of health, wealth, education, and violence prevention. These projects were conducted Kenya (42, 46) and Tanzania (48). The two Kenyan studies conducted both in slum (42) and pastoral (46) settings reported that effect of these multiple component interventions comprising violence prevention, education and health was not significantly different from that of standalone (violence prevention) intervention. Similarly, a Tanzanian study reported there was no significant difference in the rates of teen pregnancy among girls in an arm with multicomponent interventions compared to those girls in the “cash only” arm (arm that received the cash transfer program alone) (48). The multicomponent interventions comprised cash transfer conditioned up on school enrolment or seeking essential health services and unconditional cash transfer plus livelihoods and life skills training, mentoring and an asset transfer, combined with linkages to strengthened government-run HIV and SRH services).

C. Programs/Projects that addressed two components

These projects were conducted in Zambia (49, 53), Malawi (27) and Kenya (46). Some of these interventions were focused on health/social and educational support (53); some were focused on creating safe spaces and providing health vouchers, (Austrian 2020) and others were focused on educational support and livelihood support through CCT and UCCT (27). Some of these interventions were effective (46) and some were not (53).

A Kenyan study conducted in pastoral setting found that violence prevention combined with education promotion reduced teen pregnancy by one third (marginally significant); on the other hand, the effect was statistically significant for out of schoolgirls (46). Study in the same country found no significant effect of violence prevention combined with education promotion on teen pregnancy in urban informal settlement setting (42). Another Kenyan study reported that education subsidies are not effective in reducing teen pregnancy when combined with national HIV/AIDS curriculum which focuses on abstinence only messages. In addition, education subsidies when combined with an add on HIV education, and information on condom were not effective in reducing teen pregnancy (29). A Zimbabwean study conducted among urban out-of-school youth reported that combining livelihood intervention (vocational training and micro-grants) with life-skills and health education had only marginal effect on teen pregnancy when compared to life-skills and health education [HR 0.61, 95% CI, (0.37, 1.01)] (41).

Study from Zambia reported that intervention with health/social and wealth components was not effective. The study found that the provision of safe spaces for adolescents when combined with heath vouchers and heath saving accounts did not have any impact on teen pregnancy when compared to control arm (no intervention) (49).

D. Projects with one component interventions

Projects under this category include educational support (27, 37–39) and livelihood support (UCCT) (50–52), creating safe spaces for adolescents (49, 54), adolescent sexual and reproductive health education (55) and HIV prevention interventions (29, 35, 36).

Like in the case of child marriage, the study conducted in Ghana reported significant impact of provision of scholarship in reducing the probability of ever getting pregnant consistently across years (39, 40). Study conducted in Zimbabwe demonstrated that supporting orphans and vulnerable adolescents *via* School subsidies (fees, uniforms and supplies) reduced teen pregnancy both in the short term (2 years) (37) and long-term (5 years) follow ups (38). Study conducted in Malawi reported that there is no significant effect of conditional cash transfer (CCT) on marriage among schoolgirls both in short-term (2 years follow up) and long-term (5 years) follow up. The same study reported statistically significant effect of conditional cash transfer (CCT) among drop-out girls both in short-term and long-term evaluation. The study also reported that UCCT was effective on baseline schoolgirls during and immediately upon completion, but not in the long-term (after 5 years) (27).

Using data from a four-year follow up study, a Kenyan study reported that UCCT to orphan and vulnerable children significantly reduced the risk of teen pregnancy (50). On the other hand, a Malawian study reported that unconditional Cash transfer (UCCT) to ultra-poor rural households has no significant impact on teen pregnancy (52). Other multi-country study also reported that unconditional Cash transfer (UCCT) to ultra-poor rural households did not significantly reduce the risk of teen pregnancy both in Malawi and Zambia (51).

From HIV prevention projects, national HIV/AIDS curriculum that focuses on abstinence only was not effective. On the other hand, HIV prevention messages were effective in reducing teen pregnancy (29). Communicating HIV risk based on age and sex profile reduced teen pregnancy by 27.7% (*p* < 0.05). Communicating the risk averts 29.4 pregnancies by older partners. Communicating HIV risk information decreased the incidence of cross-generational pregnancies by 61.7% (relative to the comparison) (35, 36). On the other hand, a study conducted in Zimbabwe reported that community-based multi-component HIV prevention intervention does not have significant effect on teen pregnancy (56).

While school sexual and reproductive health education did not have significant impact on teen pregnancy in Tanzania (55), intensifying adolescent sexual and reproductive health services reduced teen pregnancy in Uganda (54). Another intervention with important consideration was text messaging of reproductive health information. A Ghanaian study reported that both unidirectional [odds ratio (OR) = 0.14; 95% CI = 0.03, 0.71] and interactive messages containing reproductive health information (OR = 0.15; 95% CI = 0.03, 0.86) lowered odds of self-reported pregnancy for sexually active participants (at 15 months follow up) (57).

The other important interventions under this category are creating safe spaces for adolescents. Study from Zambia reported that the provision of safe spaces for adolescents when provided alone or when combined with heath vouchers did not have any impact on teen pregnancy when compared to control arm (no intervention) (49).

## Discussions

This review attempted to search and locate the current available evidence on the effectiveness of interventions that have been implemented to reduce teen pregnancy and child marriage in sub-Saharan Africa. The interventions reported in the review were generally categorized into (a) Interventions aimed to build educational assets (E), (b) Interventions aimed to build life skills and health assets (H), (c) Interventions aimed to build livelihood and/or financial skills [Wealth building interventions (W)], (d) Interventions designed to change community norms [including violence prevention, women and girls' empowerment and/or prevention of early marriage (C) and e] Combination of one or more of the above.

Even though the amount of investment in multi-component interventions is remarkable, their (additional) effect on child marriage and teen pregnancy is not significant. For instance, multicomponent interventions comprising as many as four domains of interventions were not effective. This might have been because of the lack of focus or logistical difficulties related the implementation. The other inherent problem of studies with multiple domains of interventions is that they had multiple outcomes and they were not powered enough to detect differences in child marriage or teen pregnancy. Besides that, some lacked adequate balance at baseline. In addition, adolescent-based interventions were only subcomponents of the bigger development packages (48). The strength of such interventions, on the other hand, is that they have collected data on several health and economic variables, and it is clear to understand the modifying effect of several variables and the overall fit of the interventions within the educational, social, economic and health systems of the population. In addition, it will provide an opportunity for understanding the pathways through which the intervention affects the outcomes (child marriage and teen pregnancy). For instance, unconditional cash transfer (UCCT) (50) was effective in reducing teen pregnancy but not on reducing child marriage. From its effect on schooling, it was possible to hypothesize that the intervention reduced teen pregnancy rate not by delaying marriage, but by keeping adolescent girls in schools. Findings of the study conducted in Malawi also suggests that unconditional cash transfer programs implemented among in-school girls were only effective in the short-term (while the program was in place) (58). This may be due the fact that girls may not build assets using the short-term funding from the UCCT. On the other hand, the conditional cash transfer (CCT) was effective among out-of-school girls both in short-term and long-term follow ups and it was not effective both in short-term and long-term among in-school girls (58).

Even though it is difficult to conclude, it appears that interventions with fewer domains were promisingly effective. For instance, interventions such as education subsidies were effective when implemented alone and not effective when combined with other interventions, such as the national HIV curriculum which focuses on abstinence only prevention messages (29). School promotion interventions were effective in reducing both teen pregnancy and child marriage (29, 37–39). Particularly, study conducted in Ghana, which had a follow up period of 12 years demonstrated that provision of scholarship that covers secondary school tuition and exam fees significantly reduces marriage and pregnancy (39). Previous observational study reporting on multi-country data has also reported the effectiveness of eliminating primary school education fees in reducing child marriage (59). This underscores that addressing barriers to attending secondary school is critical not only in keeping adolescents in school but also in helping them to be empowered and delay their start of childbearing. This is in line with the findings that indicate life skills and livelihood training and mentoring interventions are effective in reducing child marriage (34, 48).

Examining the population subgroup indicates that some of the interventions were effective only among out-of-school adolescent girls, while their effect among in-school adolescent girls were not significant. For instance, study conducted in Kenya reported that the effect of the multicomponent interventions was greater among girls not in school at baseline. For instance, the intervention comprising violence prevention, education, health and wealth (VEHW) components significantly reduced child marriage compared to the control arm (violence prevention alone) among girls not in school at baseline. On the other hand, the effect was not statistically significant among the full sample (46). In addition, study conducted in Malawi found that conditional cash transfer (CCT) programs were effective both in short term and long term follow ups among out-of-school girls, but not among in-schoolgirls (58). While the mechanism is still not clear, some suggest that this may be through increasing educational outcomes (Kangwann 2022) and through tackling cost related barriers, especially among the neediest segments of the population, such as orphans (Hallfors 2015). This especially holds true for girls who could not continue schooling because of financial barriers and potentially financial barriers are really forcing girls to enter marriage early. On the other hand, some interventions, such as community dialogue were effective in reducing child marriage without impacting educational outcomes (Chow 2021). Clarifying such controversies with strong study designs might help in understanding pathways on how the interventions work.

Though routine adolescent sexual and reproductive health services were not effective (55), intensifying Youth Friendly Services (YFS) through youth corners, outreach, social and behavior change communication intervention (SBCC), counseling, family planning (60), was effective in reducing teen pregnancy. In addition, text-messages containing reproductive health information were effective (57). This is promising result as expansion of mobile technologies are increasing.

The other intervention that is promising, if implemented systematically, is community dialogue (43, 45). However, for community dialogues to be effective, there should be intensive training for the facilitators and appropriate sustainable strategy to trace the activities of community leaders. For instance, the community dialogue in Burkina Faso that used intensive training and sustained reporting and tracing mechanism was successful. On the other hand, community dialogue in Tanzania that was intense and that lacked sustained contact system was not effective (43). In addition, community dialogue has been implemented as part of other interventions (43, 45, 47). Especially, when implemented along with other interventions that are delivered through school clubs, it will increase the effect of the intervention by letting adolescent girls get adequate information to backlash misperceptions or negative interactions from the community potentially resulting from exposure to low intensity or inadequate exposure to community conversations about reducing child marriage (45).

While interpreting the effectiveness of the interventions, it is critical to take the local context into account. For instance, local security situations affect not only the effect of the interventions but also the fidelity of the interventions and the intervention uptakes. In addition, factors associated with instability (61) and migration (43) also challenge progress in the evidence base to the reduction of teen pregnancy and child marriage particularly among more vulnerable population groups in rural settings. The other critical thing that should be considered is that the effect of some interventions may cease if there is no means to accommodate their running costs and sustain them, such as the cash transfer programs (58). This implies that any innovative intervention should be sustainable, economically and politically. There is room for innovation on what might work to reduce child marriage and teen pregnancy in Sub-Saharan Africa where child marriage has remained high. In addition, because of the high demand for such interventions in fragile and humanitarian contexts, there should be an innovative mechanism to design context-specific interventions and strategies for follow up. Even though there are few studies among refugees, some were not powered for the outcome, and/or they did not include child marriage as primary outcomes (62, 63).

The current review has attempted to look broadly at evidence regarding effective interventions in reducing child marriage and teen pregnancy in sub-Saharan Africa. The review was comprehensive in addressing both published and unpublished articles. However, it is important to acknowledge certain limitations of the review. First, the review did not address studies reported in languages other than English. This is particularly a significant limitation given that Central and West Africa, which are largely Francophone, have some of the highest fertility and lowest contraceptive use levels in Africa and may have attracted interventions with fertility-related outcomes that are not published in English.

The review underscores the need for high quality research to guide program and policy options in achieving demographic transition in Africa. As described earlier, some interventions did not have adequate follow up period and their long-term effects were not investigated. Therefore, more evidence is needed to inform the design of interventions with potential to impact fertility-related outcomes in SSA.

While the findings of the review should be interpreted in the light of the above limitations, the evidence generated from the review provides clear guidance for understanding current gaps and for designing programs and policies with the potential to affect child marriage and teen pregnancy in sub-Saharan Africa.

## Conclusions

### Recommendations for research

The available evidence on the effectiveness of interventions on teen pregnancy, and child marriage is of limited quality due to the limited number of studies that have evaluated these outcomes and because of design limitations of the studies. Additionally, practical, ethical, and contextual information should be sought before implementing certain incentive-based programs. The potential for cost containment and sustainability issues should be addressed. Hence, future trials may integrate costing component. An especially important potential research agenda in Sub-Saharan Africa is exploratory qualitative studies to assess perceptions and views of various groups (urban-rural, religious groups, gender, age, policy-community leaders, etc.) on the role of financial incentives and life skills, and livelihood interventions in delaying marriage and empowering adolescents in the region. In addition, the involvement of different stakeholders, governmental and nongovernmental actors and funders is critical to get insights related to the long-term sustainability of possible interventions. Following the exploratory research, rigorous studies should be designed to delay child marriage by keeping adolescent girls in school longer and transitioning them to secondary school.

In addition, gender, and women's (and girls') empowerment initiatives are often poorly conceptualized and implemented. Research is needed to understand what these mean in high fertility settings and how such empowerment can be achieved in that context and to assess the effectiveness of such empowerment interventions on contraceptive use, pregnancy rates, fertility, and age at first marriage. Young people are key to achieving rapid and sustained decline in fertility in SSA. How to engage them meaningfully remains a challenge where further evidence is needed. Simply having youth centers has been shown not to work. Intensifying efforts to reach them with services *via* multiple strategies have been shown to be effective. There is a need to better understand strategies and mechanisms to reach adolescents and to change their fertility preference and behavior, and to empower them to make independent decisions about their lives and future, including reproductive life. Moreover, there is potential innovation room for utilizing mobile technologies to reduce teen pregnancy and child marriage.

### Recommendations for policy and practice

Emerging evidence indicates that addressing community dialogues, school subsidies and cash transfer to orphans and vulnerable groups were effective in reducing child marriage across contexts. In addition, tailored interventions, such as intensifying sexual and reproductive health services and using text messages to convey reproductive health information are effective in reducing teen pregnancy among adolescent girls. Therefore, supporting adolescent girls to stay in schools through education subsidies, and/or covering tuition and exam fees and the use of tailored sexual and reproductive health information may reduce teen pregnancies and child marriage. In addition, it is critical to use community dialogue to clarify and address cultural norms around child marriage.

## Data Availability

The original contributions presented in the study are included in the article/**Supplementary Materials**, further inquiries can be directed to the corresponding author.

## APPENDIX 1 Appraisal scores of Randomized trials.

**Table:**

S/N	Study ID	Q1	Q2	Q3	Q4	Q5	Q6	Q7	Q8	Q9	Q10	Q11	Q12	Q13	Score
1.	Asingwire 2019														10/13
2.	Austrian 2020														10/13
3.	Austrian 2022														9/13
4.	Baird 2009/10/11														10/13
5.	Baird 2015/16														10/13
6.	Bandiera 2015														10/13
7.	Bandiera 2020														10.13
8.	Cowan 2010														10/13
9.	Dake 2018														10/13
10.	Doyle 2010														10/13
11.	Duflo2006														10/13
12.	Duflo 2010														
13.	Duflo 2015														10/13
14.	Duflo 2021														9/13
15.	Dupas 2017														9/13
16.	Dunbar 2014														10/13
17.	Dupas 2011														9/13
18.	Dupas 2006														9/13
19.	Hallfors 2011														9/13
20.	Hallfors 2015														9/13
21.	Hegdahl 2021														10/13
22.	Handa 2015														9/13
23.	Kangwann 2022														10/13
24.	Rokicki 2017														10/13
25.	Sinsamala 2021														10/13
26.	UNC 2016														10/13
27.	Waidler 2022														9/13

**NB:** Q1 Was true randomization used for assignment of participants to treatment groups?

Q2 Was allocation to treatment groups concealed?

Q3 Were treatment groups similar at the baseline?

Q4 Were participants blind to treatment assignment?

Q5 Were those delivering treatment blind to treatment assignment?

Q6 Were outcomes assessors blind to treatment assignment?

Q7 Were treatment groups treated identically other than the intervention of interest?

Q8 Was follow up complete and if not, were differences between groups in terms of their follow up adequately described and analyzed?

Q9 Were participants analyzed in the groups to which they were randomized?

Q10 Were outcomes measured in the same way for treatment groups?

Q11 Were outcomes measured in a reliable way?

Q12 Was appropriate statistical analysis used?

Q13 Was the trial design appropriate, and any deviations from the standard RCT design (individual randomization, parallel groups) accounted for in the conduct and analysis of the trial?

## APPENDIX 2 Appraisal scores of quasi-experimental studies.

**Table:**

S/N	Study ID	Q1	Q2	Q3	Q4	Q5	Q6	Q7	Q8	Q9	Score
1.	Chow 2021										8/9
2.	Eruulkar 2009										8/9
3.	Erulkar 2020										

**NB:**

Q1 Is it clear in the study what is the “cause” and what is the “effect” (i.e., there is no confusion about which variable comes first)?

Q2 Were the participants included in any comparisons similar?

Q3 Were the participants included in any comparisons receiving similar treatment/care, other than the exposure or intervention of interest?

Q4 Was there a control group?

Q5 Were there multiple measurements of the outcome both pre and post the intervention/exposure?

Q6 Was follow up complete and if not, were differences between groups in terms of their follow up adequately described and analyzed?

Q7 Were the outcomes of participants included in any comparisons measured in the same way?

Q8 Were outcomes measured in a reliable way?

Q9 Was appropriate statistical analysis used?

## APPENDIX 3 Full text studies excluded with reasons.

**Table:**

No.	Study	Reason for exclusion
1)	Ampt 2020	This has addressed commercial sex workers regardless of age
2)	Siaplay 2012	This is a longitudinal follow up study that used secondary data. It had higher attrition rate. overall response rates of young adults in waves 2, 3, and 4 were 83%, 74%, and 72%, respectively.
3)	Heinrich 2016	No comparison group
4)	Oberth 2021	The design was observational (cohort)
5)	Koski-2018	The design was observational (used DHS data for comparison)
6)	Ingwersen 2019	No comparison group
7)	Hand 2015b	Population covered was women of reproductive age (child marriage and teen pregnancy were not covered), but the study addressed TFR, pregnancy rate, etc.
8)	Buehren 2017	Average age was 22 years (older women). More than a third of participants were already married and had at least 1 child
9)	Buehren 2017a	There are women who are already in marital union and who had children before the intervention and that proportion is different across treatment arms
10)	Miller 2013	This is a costing study that considers costs saved by averting HIV infections because of prevention of marriage and increasing income by keeping them in school
11)	Erulkar 2017	This a costing study
12)	Stark 2018a	Child marriage for girls already married at baseline was included. The study was not powered for child marriage
13)	Stark 2018B	Child marriage was not primary outcome
14)	Sarnquist 2016	Reports pregnancy related dropouts, but not total pregnancies
15)	Gavrilovic 2020	This is a single time cross-sectional study assessing the impact of productivity safety net program (PSNP) on child marriage rate. The child marriage was estimated by asking women (aged 12–19) in the households who have got married before the age of 18 in the last five years or in their lifetime. However, it is difficult to attribute these changes to the intervention because the measurement does not precisely coincide with the timing of the introduction of the intervention. In addition, comparability of the comparison sites was not measured before the introduction of the intervention. The comparative analysis for the two interventions dos does not show effect of the interventions, because comparison between Integrated safety net program (ISNP) and PSNP was made before the ISNP was rolled out.
16)	Palermo 2018	Baseline imbalance with respect to marriage rates. The design was not focused (it is part of multiple evaluation projects ranging from food consumption shares and other basic needs. The analysis was based on RDD and rather than priori pseudo or true assignment to control and intervention group. The content and detailed description of the intervention specifically targeting adolescents is not clear. In addition, because of lack of priori planning there was no follow up of cohorts of girls, but only estimation of marriage rates in the population, which is further challenging to infer to the effect of the intervention in such design.
17)	Austrian 2021	The report addressed the effect of the intervention on education, health and economic outcomes, but not on pregnancy and marriage
18)	Taylor 2014	The report includes the effect of the intervention on attitudinal factors, not changes in marriage and teen pregnancy
