# Supplementary material for: Effectiveness of interventions to reduce child marriage and teen pregnancy in sub-Saharan Africa: A systematic review of quantitative evidence
Source: Front Reprod Health. 2023 Mar 31;5:1105390. doi: 10.3389/frph.2023.1105390 (PMC10103588; doi:10.3389/frph.2023.1105390)
Supplement: Supplementary file 1 [file Table1.docx]

# Supplementary file 1: Search strategy

1. **PubMed**

Last search date: January 26, 2022

| **S/n** | **Search** | **Results** |
| --- | --- | --- |
| 1 | "Birth Rate"[Mesh] OR "Pregnancy in Adolescence"[Mesh] OR "Pregnancy Rate"[Mesh] OR "Marriage"[Mesh] | 52,030 |
| 2 | Adolescent birth [tw] OR teen pregnancy [tw] OR adolescent pregnancy OR early marriage [tw] OR Adolescent marriage [tw] | 100,148 |
| 3 | "Africa South of the Sahara"[Mesh] | 234,671 |
| 4 | Cameroon[Mesh] OR Chad[Mesh] OR Congo[Mesh] OR "Equatorial Guinea"[Mesh] OR Gabon[Mesh] OR Burundi [Mesh] OR Djibouti[Mesh] OR Eritrea[Mesh] OR Kenya[Mesh] OR Ethiopia[Mesh] OR Rwanda [MESH] OR Somalia [MESH] OR "South Sudan" [MESH] OR Sudan [MESH] OR Tanzania [MESH] OR Uganda [MESH] OR Angola [MESH] OR Botswana [MESH] OR Lesotho [MESH] OR Mozambique [MESH] OR Namibia [MESH] OR "South Africa" [MESH] OR Swaziland [MESH] OR Eswatini [MESH] OR Zambia [MESH] OR Zimbabwe [MESH] OR Benin [MESH] OR "Burkina Faso" [MESH] OR "Cabo Verde" [MESH] OR Gambia [MESH] OR Ghana [MESH] OR Guinea [MESH] OR “Guinea-Bissau” [MESH] OR Liberia [MESH] OR Mali [MESH] OR Mauritania [MESH] OR Niger [MESH] OR Nigeria [MESH] OR Senegal [MESH] OR “Côte d'Ivoire” [MESH] OR “Sierra Leone" [MESH] | 205,721 |
| 5 | "Sub-Saharan" [tw] OR Africa [tw] OR Cameroon [tw] OR Chad [tw] OR Congo [tw] OR “Equatorial Guinea” [tw] OR Gabon [tw] OR Sao Tome and Principle [tw] OR Burundi [tw] OR Djibouti [tw] OR Eritrea [tw] OR Kenya [tw] OR Ethiopia [tw] OR Rwanda [tw] OR Somalia [tw] OR “South Sudan” [tw] OR Sudan [tw] OR Tanzania [tw] OR Uganda [tw] OR Angola [tw] OR Botswana [tw] OR Lesotho [tw] OR Mozambique [tw] OR Namibia [tw] OR “South Africa” OR Swaziland OR Eswatini OR Zambia [tw] OR Zimbabwe [tw] OR Benin [tw] OR “Burkina Faso” [tw] OR “Cabo Verde” OR Gambia [tw] OR Ghana [tw] OR Guinea [tw] OR Guinea-Bissau [tw] OR Liberia [tw] OR Mali [tw] OR Mauritania [tw] OR Niger [tw] OR Nigeria [tw] OR Senegal [tw] OR Côte d'Ivoire OR Ivory Coast Sierra Leone [tw] OR Togo [tw] | 592,691 |
| 6 | 1 OR 2 | 141,270 |
| 7 | 3 OR 4 OR 5 | 597,998 |
| 8 | 6 AND 7 | 12,182 |
| 9 | limit 8 to Clinical Trial, Clinical Trial, Phase II, Clinical Trial, Phase III, Clinical Trial, Phase IV, Evaluation Study, Pragmatic Clinical Trial, Randomized Controlled Trial, Humans, English, Adolescent: 13-18 years | 738 |
| 10 | limit to15-18 adolescents | 704 |

1. **CINAHL**

Last search January 17, 2022

| **S/n** | **Search** | **Results** |
| --- | --- | --- |
|  | (MH "Pregnancy in Adolescence+") OR (MH "Pregnancy, Unplanned") OR (MH "Marriage") OR (MH "Maternal Age 14 and Under") | 1,7034 |
|  | TI (“adolescent birth” OR “teen pregnancy” OR “adolescent pregnancy” OR “early marriage”) | 792 |
|  | AB (“adolescent birth” OR “teen pregnancy” OR “adolescent pregnancy” OR “early marriage”) | 1,303 |
|  | (MH "Africa South of the Sahara+") | 77,200 |
|  | TI ("Sub-Saharan" OR "Africa) OR Cameroon OR Chad OR Congo OR Equatorial guinea OR Gabon OR Sao Tome and Principle OR Burundi OR Djibouti OR Eritrea OR Kenya OR Ethiopia OR Rwanda OR Somalia OR South Sudan OR Sudan OR Tanzania OR Uganda OR Angola OR Botswana OR Lesotho OR Mozambique OR Namibia OR South Africa OR Swaziland OR Eswatini OR Zambia OR Zimbabwe OR Benin OR Burkina Faso OR Cabo Verde OR Gambia OR Ghana OR Guinea OR Guinea-Bissau OR Liberia OR Mali OR Mauritania OR Niger OR Nigeria OR Senegal OR Côte d'Ivoire OR Ivory Coast OR Sierra Leone OR Togo) | 99,729 |
|  | AB ("Sub-Saharan" OR "Africa) OR Cameroon OR Chad OR Congo OR Equatorial guinea OR Gabon OR Sao Tome and Principle OR Burundi OR Djibouti OR Eritrea OR Kenya OR Ethiopia OR Rwanda OR Somalia OR South Sudan OR Sudan OR Tanzania OR Uganda OR Angola OR Botswana OR Lesotho OR Mozambique OR Namibia OR South Africa OR Swaziland OR Eswatini OR Zambia OR Zimbabwe OR Benin OR Burkina Faso OR Cabo Verde OR Gambia OR Ghana OR Guinea OR Guinea-Bissau OR Liberia OR Mali OR Mauritania OR Niger OR Nigeria OR Senegal OR Côte d'Ivoire OR Ivory Coast OR Sierra Leone OR Togo) | 103,354 |
|  | 1 OR 2 OR 3 | 17,491 |
|  | 4 OR 5 OR 6 | 109,943 |
|  | 7AND 8 | 1,217 |
|  | Narrow by Subject Geographic: - Africa; Narrow by Subject Age: - adolescent: 13-18 years, Narrow by Language: - English | 141 |
|  | Limit 17 to Randomized controlled trials and Exclude MEDLINE records | 0 |

1. **EMBASE**

| **S/n** | **Search** | **Results** |
| --- | --- | --- |
|  | 'adolescent birth':ti,ab,kw OR 'teen pregnancy':ti,ab,kw OR 'adolescent pregnancy':ti,ab,kw OR 'early marriage':ti,ab,kw | 3,708 |
|  | 'Adolescent pregnancy'/exp | 9,991 |
|  | 'Africa south of the Sahara'/exp | 285,856 |
|  | ('sub-saharan':ti,ab,kw OR africa:ti,ab,kw OR cameroon:ti,ab,kw OR chad:ti,ab,kw OR congo:ti,ab,kw OR 'equatorial guinea':ti,ab,kw OR gabon:ti,ab,kw OR 'sao tome':ti,ab,kw) AND principle:ti,ab,kw OR burundi:ti,ab,kw OR djibouti:ti,ab,kw OR eritrea:ti,ab,kw OR kenya:ti,ab,kw OR ethiopia:ti,ab,kw OR rwanda:ti,ab,kw OR somalia:ti,ab,kw OR 'south sudan':ti,ab,kw OR sudan:ti,ab,kw OR tanzania:ti,ab,kw OR uganda:ti,ab,kw OR angola:ti,ab,kw OR botswana:ti,ab,kw OR lesotho:ti,ab,kw OR mozambique:ti,ab,kw OR namibia:ti,ab,kw OR 'south africa':ti,ab,kw OR swaziland:ti,ab,kw OR eswatini:ti,ab,kw OR zambia:ti,ab,kw OR zimbabwe:ti,ab,kw OR benin:ti,ab,kw OR 'burkina faso':ti,ab,kw OR 'cabo verde':ti,ab,kw OR gambia:ti,ab,kw OR ghana:ti,ab,kw OR guinea:ti,ab,kw OR 'guinea bissau':ti,ab,kw OR liberia:ti,ab,kw OR mali:ti,ab,kw OR mauritania:ti,ab,kw OR niger:ti,ab,kw OR nigeria:ti,ab,kw OR senegal:ti,ab,kw OR 'côte divoire':ti,ab,kw OR 'ivory coast':ti,ab,kw OR 'sierra leone':ti,ab,kw OR togo:ti,ab,kw | 371,906 |
|  | 1 OR 2 | 11,431 |
|  | 4 OR 5 | 462,050 |
|  | 5 AND 6 | 939 |
|  | Limit 7 to ('clinical trial'/de OR 'comparative effectiveness'/de OR 'controlled clinical trial'/de OR 'intervention study'/de OR 'randomized controlled trial'/de OR 'randomized controlled trial topic'/de) AND [adolescent]/lim AND [humans]/lim AND [english]/lim AND [embase]/lim | 25 |

1. **Cochrane CENTRAL**

Last date run: 01/26/2021

| ID | Search | Hits |
| --- | --- | --- |
| #1 | MeSH descriptor: [Pregnancy in adolescence] explode all trees | 215 |
| #2 | "Adolescent birth” OR “teen pregnancy” OR “adolescent pregnancy” OR “early marriage” (Word variations have been searched) | 6,356 |
| #3 | Sub-Saharan Africa | 1,861 |
| #4 | MeSH descriptor: [Africa South of the Sahara] explode all trees | 7151 |
| #5 | #1 OR #2 | 5,632 |
| #6 | #3 OR #4 | 8373 |
| #8 | #8 AND #9 | 490 |
